# Obesity among Scottish 15 year olds 1987–2006: prevalence and associations with socio-economic status, well-being and worries about weight

**DOI:** 10.1186/1471-2458-8-404

**Published:** 2008-12-09

**Authors:** Helen Sweeting, Patrick West, Robert Young

**Affiliations:** 1 MRC Social and Public Health Sciences Unit, 4, Lilybank Gardens, Glasgow, G12 8RZ, UK

## Abstract

**Background:**

Increases in the prevalence of child and adolescent obesity have accelerated since the mid 1980s. Socio-economic status (SES)-adiposity relationships appear less clear in adolescence than childhood, and evidence on whether increasing obesity is itself patterned according to SES is inconsistent. Increasing prevalence may have increased the tolerance, and reduced recognition of, or concern about, obesity. The aim of this study is to report the prevalence of obesity and its association with SES, well-being and worries about weight among 15-year olds in 1987, 1999 and 2006.

**Methods:**

Height and weight data obtained from 15-year olds in 1987 (N = 503), 1999 (N = 2,145) and 2006 (N = 3,019), allowed categorisation of obesity (UK90 criteria). SES was represented by parental occupational class and area deprivation; psychological wellbeing by the 12-item General Health Questionnaire (GHQ-12) and self-esteem; weight worries by 'a lot' of worry about weight.

**Results:**

Obesity prevalence was 6.7%, 10.6% and 15.9% (males), and 5.4%, 11.5% and 14.9% (females) in 1987, 1999 and 2006. Among obese males, BMIs increased over time. There was little evidence of differentials in obesity in respect of either SES measure, and none for increased disparities over time. There was no association between obesity and GHQ-12 'caseness' or (except females in 2006) self-esteem. Weight worries were more prevalent among the obese and increased over time overall, but the obesity-weight worry relationship did not change. At each date, large proportions of the obese *did not *worry 'a lot' about weight, while among the non-obese, up to 18.8% males and 40.1% females (in 2006) *did *worry.

**Conclusion:**

Between 1987 and 2006, prevalence of obesity among Scottish 15 year olds increased around 2.5 times. However, this increasing prevalence did not impact on the obesity-weight-worry relationship. While many obese adolescents appear unconcerned about their weight, a significant minority of the non-obese worry needlessly.

## Background

Obesity has been described as the most common paediatric disease in most of the world [1]. In the UK, as much of Europe, increases in the prevalence of child and adolescent overweight and obesity accelerated over the two decades from the mid 1980s [2,3]. In addition, the distribution of body mass index (BMI) has become more skewed, increasing most at the highest levels [4-6].

In respect of the relationship between adiposity and socio-economic status (SES), a review focusing on studies conducted in developed countries between 1990 and 2005, found the majority of associations were inverse, although in many the relationship was not strictly linear and was clearer in childhood than adolescence. This review also called for studies to use more than one SES indicator, since different patterns in respect of parental and neighbourhood SES might indicate different causal mechanisms [7]. For example, a large Canadian study found independent effects of both parental education and neighbourhood SES on overweight among 5–17 year olds [8]. In respect of time-trends, the results of the few studies to examine whether the increasing prevalence is itself patterned according to SES are inconsistent. Thus, while some have found the greatest increases among those from the lowest income families in the US [9] and UK [2], another US study suggested that income-based disparities have weakened over time [10].

Evidence in respect of the psychological consequences of child and adolescent obesity is also mixed, some studies concluding that obesity is associated with psychological or psychiatric problems [11], others finding no differences in self-esteem [12], depression or anxiety [13]. The majority suggest that while obese community samples have lower body satisfaction than non-obese, few are depressed or have low global self-esteem [14], even in population sub-groups where appearance and slimness are most valued [15]. Psychological distress appears to be more strongly associated with concern about weight and shape, regardless of BMI [16,17].

A potential reason for such inconsistent findings is that increasing prevalence means "overweight is the new 'normal' weight" in many countries [18]. In Scotland, around 60% of adults were overweight or obese in 2003 [19]. Although it is possible that failure to achieve the ideal Western lean body image may have led to inadequacy and guilt among an increasing proportion of the population [20], an alternative is that the upward trend may have reduced stigmatisation and increased tolerance of obesity [21], and made overweight more difficult to recognise [18,22]. For example, comparison of two surveys of British adults conducted in 1999 and 2007, found that despite increasing BMIs, the threshold at which people perceived themselves as overweight increased, thus reducing the proportion who correctly identified themselves as overweight [22]. Similarly, the proportions of both overweight and normal weight Finnish adolescents who perceived themselves as overweight reduced between 1979 and 1999 [23]. Among 11–14 year olds in London in 2001, around 60% overweight and 30% obese boys believed their weight to be 'about right'; equivalent figures among girls were 34% and 9% [24]. A qualitative study, conducted among Scottish 13–14 year olds from disadvantaged areas, found that among the overweight and obese, acceptance of body size was common [25].

This paper draws on data from three cohorts of 15-year olds in the final year of statutory mainstream education (Scottish Secondary 4), resident in the Central Clydeside Conurbation (a predominantly urban area centred around Glasgow in the West of Scotland) in 1987, 1999 and 2006. The aims are to: (1) present data on BMI and obesity at each date; (2) examine the patterning of obesity according to an individual (social class) and area-based (Carstairs deprivation category) measure of SES at each date and explore the possibility of differential increases in the prevalence of obesity among different SES groups; and (3) determine the association between obesity and psychological distress (GHQ-12 'caseness'), self-esteem and worries about weight at each date.

## Methods

### Samples

Data are drawn from 15-year olds in their final year of mainstream statutory education (S4), who participated in the 'West of Scotland Twenty-07 Study: Health in the Community' (***'Twenty-07' ***[26,27]), the 'West of Scotland 11 to 16 Study: Teenage Health' (***'11 to 16' ***[28-30]) or the most recent study in the series, 'Peers and Levels of Stress' (***'PaLS' ***[31]). All three studies received approval from research ethics committees at the University of Glasgow.

***'Twenty-07' ***included a youth cohort, first surveyed in 1987 at age 15. A response rate of 65% of the issued sample was obtained; examination of bias due to non-response revealed no significant gender or social class differences compared with the population [32]. Data were obtained from both the young person and (separately) a parent via home interviews, self-complete questionnaires and physical measures. To maintain comparability with the two later studies, only respondents who were in the (mainstream) S4 school year at the time they were weighed and measured were eligible for inclusion in the analyses (N = 505); those who were in S5 (N = 307), had left school (N = 150), were attending a special school (N = 4) or missing in respect of all physical measures (N = 43) were excluded; restricting the sample in this way shifts the SES distribution slightly upwards. Of the 505 eligible for comparison, height and weight data are available for 503, mean age 15 years 8 months, 48% males.

***'11 to 16' ***was a school-based study of a cohort in mainstream education. The sampling scheme involved a number of steps to ensure its representativeness [33]. At the second follow-up in 1999, 2,196 respondents, in S4 classes in 43 secondary schools, took part. This group represented 85% of the baseline and 79% of the original eligible samples; height and weight data are available for 2,145, mean age 15 years 5 months, 51% males. During classroom sessions, respondents completed questionnaires and were weighed and measured. Comparison with census data showed the baseline sample to be representative of the population from which it was drawn in respect of gender and social class; thereafter, differential attrition (e.g. persistent school truants) made it less so [34].

***'PaLS'***, conducted in 2006, was also mainstream school-based. The sampling scheme aimed to obtain a representative sample, and within selected schools, all pupils in the S4 year group were invited to participate. The total sample comprised 3,194 respondents, representing 81% of the eligible sample; height and weight data are available for 3,019, mean age 15 years 5 months, 50% males. Respondents completed questionnaires and were weighed and measured. Participating schools did not differ significantly from the remainder in the area in respect of a number of socio-demographic dimensions, nor pupil achievement by the end of statutory schooling. However within selected schools, those pupils who completed a questionnaire and were weighed and measured lived in less deprived areas than non-responders [31].

### Measures

#### Obesity

All height and weight measurements were taken in indoor clothes with no footwear. BMI (kg/m^2^) was converted into standard deviation scores compared to the UK 1990 growth reference [35], those above the 95^th ^percentile for age and sex being defined as obese.

#### SES

***Social class ***was defined on the basis of the occupation of the head of the household. In the 1987 study, this information was obtained via parental interview, in 1999 via parental self-completion questionnaire (collected during an earlier wave, when the study pupils were aged 11) supplemented, where necessary, by information provided by the pupils during interviews with the nurses [36], and in 2006 entirely via pupil interview. In these analyses, social class was categorised into four categories: non-manual (comprising Class I, professional; Class II, managerial and technical; and Class IIINM, skilled non-manual occupations); Class IIIM (skilled manual); Classes IV (semi-skilled) and V (unskilled); and missing. ***Carstairs-Morris deprivation categories ***[37] were assigned to home postcodes, pupil postcode information being supplemented by data from schools in the (schools-based) 1999 and 2006 studies. This area-based measure reflects access to goods and services, resources and amenities [37]; in these analyses it was collapsed into low (categories 1–3), mid (4–5), high (6–7) and missing deprivation categories.

#### Psychological well-being and weight worries

Respondents completed the shortest, ***12-item General Health Questionnaire (GHQ-12)***. This measure has been validated for use with both younger [38] and older [39] adolescents. The GHQ was designed as a measure of state, focussing on inability to carry out normal functions (e.g. 'been able to enjoy your normal day-to-day activities') and the emergence of distressing symptoms (e.g. 'felt constantly under strain'). Each item includes four answer options which can be scored in several ways. Traditional, binary scoring indicates deviations from normal (0-0-1-1), and GHQ 'caseness' is generally defined via thresholds based on this method [40]; the standard cut-off for GHQ-12 'caseness' is 2/3 [39,40]. A 10 item ***self-esteem scale ***(based on Rosenberg [41], with items such as 'I am pretty sure of myself', 'I often wish I was someone else') was included at each date, summed to produce a total score. Total scores were not comparable across the studies, since the 1999 and 2006 studies used a 4-point scale (strongly agree-strongly disagree), but the 1987 study included an additional mid-point. Analyses therefore used a dichotomous measure, defining the lowest quartile in each study as having 'low self-esteem'. A measure of ***worries about weight ***was derived from checklists of personal concerns (e.g. 'How much do you worry about doing well at school?' '... about how your family gets on with each other?') which were included in each study. In 1987 and 1999, respondents were asked how much they worried about 'weight', but in 2006, two weight-related worry items were included, 'being overweight' and 'being too thin'. Since it is possible that some endorsing the weight item in the two earlier studies were worried about being underweight, a 'weight worries' variable was defined as those reporting 'a lot' of worry about 'weight' (1987 and 1999) and about either 'overweight' or 'thin' (2006).

### Analyses

Analyses were performed separately for males and females. Analyses of differences in BMI (according to date) used the F-test, those of obesity (according to date and SES at each date) X^2^, and those of GHQ-12 'caseness', 'low' self-esteem and weight worries' (according to obesity at each date), logistic regression.

Probabilistic weights have been constructed to compensate for differential attrition in the ***'11 to 16' ***(1999) Study [34] and for socio-demographic differences between responders and non-responders in the ***'PaLS' ***(2006) Study [31]. However, since the results in respect of both prevalence of obesity and associations with SES and well-being using weighted and unweighted data were very similar, those based on unweighted data are presented here.

## Results

Basic descriptive statistics for males and females at each date are shown in Table 1 [see Additional file 1]. Mean BMIs in 1987, 1999 and 2006 were 20.4, 20.7 and 21.4 for males (F = 17.0, p = .000), and 21.0, 21.5 and 22.1 for females (F = 15.9, p = .000). Male BMI increases over the 12 year period from 1987 to 1999 (F = 1.6, p = .206) were therefore somewhat smaller than those over the 7 year period from 1999 to 2006 (F = 24.7, p = .000). The prevalence of male obesity increased from 6.7% in 1987 to 10.6% in 1999 and 15.9% in 2006 (earlier increase p = .067; later increase p = .000). Corresponding rates for females were 5.4%, 11.5% and 14.9% (earlier p = .003; later p = .014). Thus, for males, the increase in prevalence of obesity, like BMI, was steeper over the 1999–2006 period. In addition (not shown on table), among *obese *males, mean (SD) BMIs also increased over time, from 26.2 (1.1) in 1987 to 27.0 (2.2) in 1999 and 28.3 (2.9) in 2006 (F = 12.4, p = .000). No such increase was seen among obese females, among whom mean (SD) BMIs were 28.4 (3.1), 28.7 (2.7) and 28.7 (2.8) in 1987, 1999 and 2006 (F = 0.1, p = .932).

Changes in SES reflect those in society over this period [42], with some minor variations in area deprivation. GHQ 'caseness' increased significantly among females over the earlier time period (p = .000) and among both males and females over the later one (p = .000 for both genders) (see also [43]). A clear gender difference in 'low self-esteem' can be seen at each date, but because this variable was defined as those with scores in the lowest quartile in each study, changes over time cannot be identified. Finally, the proportion reporting 'a lot' of weight worries also increased over time. The gender difference was greatest in 1987 (6.2% males worried 'a lot', compared with 29.0% females), and increases between 1987 and 1999 were greater for males, but significant for both genders (male p = .000; female p = .005). Thereafter, weight worries remained stable among males, but increased slightly among females (p = .005).

Table 2 [see Additional file 2] shows associations between obesity and both social class and area deprivation category, for males and females at each date. There were no significant social class differences in obesity at any date, for either males or females, and no differences according to area deprivation among males. However, among females, there were significant differences according to deprivation in 1987 (p = .000) and 2006 (p excluding 'missing' category = .035). At both dates, lowest prevalence occurred among those resident in less deprived areas, although at the later date the association was not linear.

Finally, the table shows that obesity increased between 1987 and 2006 for all gender and social class and area deprivation groups, apart from females from the most deprived areas.

Table 3 [see Additional file 3] shows that, apart from significantly increased odds of 'low' self-esteem among obese compared with non-obese females in 2006, neither GHQ-12 'caseness' nor 'low' self-esteem was associated with obesity at any date for either males or females (see also [30]).

At each date, obese males were around twice as likely as their non-obese peers to report weight worries, although wide CIs mean this was not significant in 1987. A trend towards a weakening relationship between obesity and male weight worries over time was not significant. In 1987 and 2006, but not 1999, obese females were, like males, around twice as likely as their non-obese peers to report weight worries. Despite the rather different association in 1999, the date by obesity interaction for female weight worries was not significant. At each date, considerable proportions of the obese *did not *report 'a lot' of worry about weight (e.g. 71.2% males, 40.2% females in 2006), while a significant minority of the non-obese *did *report 'a lot' of worry (e.g. 18.8% males, 40.1% females in 2006).

## Discussion

This paper has examined trends in obesity, based on data from three samples identical in respect of age, school year and geographical location. Although the smaller sample size and lower prevalence of obesity at the earliest date means that confidence intervals for the 1987 data are wide, the strength of the study lies in the availability of equivalent, or near equivalent, measures of SES and psychological well-being at each date.

In 1987, 6.7% male and 5.4% female 15 year olds in the West of Scotland were obese. Given that the definition of obesity used here was BMIs above the 95^th ^percentile for age and sex of the UK 1990 growth reference [35], the 1987 prevalence was already high, and consistent with other studies finding higher prevalence among children and adolescents in Scotland compared with England [44]. Over the following 19 year period to 2006, prevalence increased around 2.5 times.

Among females, increases in both mean BMIs and prevalence of obesity appeared steady, however, no increase was seen among mean BMIs among those categorised as obese (i.e. the upper tail of the distribution). In contrast, consistent with studies conducted in the UK and elsewhere (e.g. [2-6]) among males, more recent (1999–2006) increases were steeper, and mean BMIs among those at the upper end of the distribution increased significantly. Thus, while the prevalence of obesity increased among both males and females, it was only among males that the obese got heavier.

Obesity was not differentiated according to social class, an individual level SES measure, at any date. Differentiation according to area level deprivation was apparent among females in 1987 and 2006, but the clear pattern of (much) lower prevalence among those from the least, and higher levels among those from the most deprived areas in 1987 was not seen in 2006. These results provide little evidence for higher prevalence of adolescent obesity with falling SES, nor for increased disparities over time. If anything, among females, and consistent with one US study [10], disparities appear to have weakened. This lack of differentiation in obesity according to SES is consistent with several other studies of adolescents [7], and is interesting in view of other findings indicating that in contrast to the earlier stage of childhood and the later stage of adulthood, the situation in youth is "characterised more by the absence then presence of class variation" [45]. This has been seen in a number of specific studies in both the UK [46] and elsewhere [47], and in respect of a broad range of UK datasets, where, with the important exception of severe chronic illness, most morbidity measures, including less severe conditions, symptoms, non-fatal accidents and certain dimensions of mental health, show evidence of a reduction in class patterning between childhood and youth [48]. Analyses of both our ***'Twenty-07' ***(1987) [27] and ***'11 to 16' ***(1999) [29] studies also found little evidence of class variation on a number of health indicators. These ranged from subjective assessments to objective physical measures, with the exception of height which, as an indicator of health potential [49], is patterned by SES from the earliest years.

Associations between obesity and both GHQ-12 'caseness' and 'low' self-esteem were small and generally non-significant. However, again consistent with the majority of other studies [14], the obese were more concerned about their weight. Of interest was whether relationships between any of these measures of psychological well-being and obesity had changed over time, particularly whether they might have weakened as the prevalence of obesity increased. There was no evidence of this; rather, weight worries increased among both the obese and non-obese. Perhaps more striking is the proportion of the obese who did *not *report 'a lot' of worry about weight, consistent with other studies suggesting failure to recognise obesity and/or acceptance of body size among UK adolescents [24,25].

## Conclusion

Prevalence of obesity among 15 year olds in the West of Scotland was above that of the UK as a whole in 1987, and in the following 19 year period it increased significantly. An accelerating increase in prevalence, and greater BMI increases at the highest levels were seen in males but not females. There was little evidence of socio-economic differentials in prevalence, consistent with a number of other studies of adolescent obesity and ill-health, nor of significantly increased levels of psychological distress among the obese at any of the three dates studied.

The proportion reporting worries about weight increased most between 1987 and 1999, thereafter changing little, despite continuing increases in obesity levels. This in itself might suggest that, as others have noted, overweight and obesity are increasingly unrecognised. However, if that were the case, our analyses would have shown a weakening relationship between obesity and weight worries, and they did not. Instead, they suggest that although obese adolescents are more likely to be concerned about their weight than their non-obese peers, large proportions of the obese (particularly males) do not appear overly concerned about their weight, while many non-obese (particularly females) worry needlessly.

## Competing interests

The authors declare that they have no competing interests.

## Authors' contributions

HS devised and conducted the analyses reported here, and drafted the manuscript. PW and RY commented on the analyses and each draft, and all authors read and approved the final manuscript. PW contributed to the data collection in all three studies, HS to the ***'11 to 16' ***and ***'PaLS' ***studies, and RY to ***'PaLS'***.

## Pre-publication history

The pre-publication history for this paper can be accessed here:



## Supplementary Material

Additional file 1 **Table 1: Descriptive statistics – males and females at each date.** Click here for file

Additional file 2 **Table 2: Associations between obesity and socio-economic status (social class and area deprivation) – percentages (95% CIs) (and numbers) of males and females at each date.** Click here for file

Additional file 3 **Table 3: GHQ 'caseness', 'low' self-esteem and weight worries by obesity status – males and females at each date.** Click here for file
