# Supplementary material for: Obesity among Scottish 15 year olds 1987–2006: prevalence and associations with socio-economic status, well-being and worries about weight
Source: BMC Public Health. 2008 Dec 9;8:404. doi: 10.1186/1471-2458-8-404 (PMC2615437; doi:10.1186/1471-2458-8-404)
Supplement: Additional file 1 — Table 1: Descriptive statistics – males and females at each date. [file 1471-2458-8-404-S1.doc]

**Table 1: Descriptive statistics – males and females at each date.**

|  |  |  |  |  |  |  |  |  |  |  |  |  |
| --- | --- | --- | --- | --- | --- | --- | --- | --- | --- | --- | --- | --- |
|  | **Males** | | | | | | **Females** | | | | | |
|  |  |  |  |  |  |  |  |  |  |  |  |  |
|  | **1987** | | **1999** | | **2006** | | **1987** | | **1999** | | **2006** | |
|  |  |  |  |  |  |  |  |  |  |  |  |  |
|  | **Mean** | **(SD)** | **Mean** | **(SD)** | **Mean** | **(SD)** | **Mean** | **(SD)** | **Mean** | **(SD)** | **Mean** | **(SD)** |
|  |  |  |  |  |  |  |  |  |  |  |  |  |
| **BMI** | 20.4 | (2.5) | 20.7 | (3.0) | 21.4 | (3.8) | 21.0 | (3.0) | 21.5 | (3.3) | 22.1 | (3.7) |
|  |  |  |  |  |  |  |  |  |  |  |  |  |
|  | **N** | **(%)** | **N** | **(%)** | **N** | **(%)** | **N** | **(%)** | **N** | **(%)** | **N** | **(%)** |
| **Obese** |  |  |  |  |  |  |  |  |  |  |  |  |
| Yes | 16 | (6.7) | 116 | (10.6) | 242 | (15.9) | 14 | (5.4) | 121 | (11.5) | 223 | (14.9) |
| No | 223 | (93.3) | 978 | (89.4) | 1279 | (84.1) | 247 | (94.6) | 930 | (88.5) | 1275 | (85.1) |
| Missing | 2 |  | 22 |  | 51 |  | 3 |  | 29 |  | 124 |  |
| **Social class** |  |  |  |  |  |  |  |  |  |  |  |  |
| Non-manual | 122 | (50.8) | 507 | (48.4) | 869 | (62.7) | 116 | (44.6) | 459 | (46.5) | 794 | (58.5) |
| III-manual | 75 | (31.3) | 334 | (31.9) | 326 | (23.5) | 97 | (37.3) | 299 | (30.3) | 344 | (25.3) |
| IV-V | 43 | (17.9) | 206 | (19.7) | 192 | (13.8) | 47 | (18.1) | 230 | (23.3) | 220 | (16.2) |
| Missing | 1 |  | 69 |  | 185 |  | 4 |  | 92 |  | 264 |  |
| **Area deprivation category** |  |  |  |  |  |  |  |  |  |  |  |  |
| Low (categories 1-3) | 107 | (44.4) | 410 | (40.0) | 663 | (42.4) | 110 | (41.7) | 378 | (36.8) | 620 | (38.3) |
| Mid (categories 4-5) | 97 | (40.2) | 323 | (31.5) | 592 | (37.9) | 105 | (39.8) | 345 | (33.6) | 648 | (40.1) |
| High (categories 6-7) | 37 | (15.4) | 291 | (28.4) | 307 | (19.7) | 49 | (18.6) | 304 | (29.6) | 349 | (21.6) |
| Missing | 0 |  | 92 |  | 10 |  | 0 |  | 53 |  | 5 |  |
| **GHQ caseness** |  |  |  |  |  |  |  |  |  |  |  |  |
| Yes | 30 | (12.7) | 164 | (15.1) | 323 | (21.5) | 48 | (18.8) | 344 | (32.5) | 678 | (44.1) |
| No | 207 | (87.3) | 921 | (84.9) | 1182 | (78.5) | 208 | (81.3) | 713 | (67.5) | 861 | (55.9) |
| Missing | 4 |  | 31 |  | 67 |  | 8 |  | 23 |  | 83 |  |
| **‘Low’ self-esteem** |  |  |  |  |  |  |  |  |  |  |  |  |
| Yes | 32 | (14.5) | 135 | (12.4) | 196 | (13.1) | 81 | (32.1) | 338 | (32.4) | 567 | (36.8) |
| No | 188 | (85.5) | 957 | (87.6) | 1297 | (86.9) | 171 | (67.9) | 705 | (67.6) | 974 | (63.2) |
| Missing | 21 |  | 24 |  | 79 |  | 12 |  | 37 |  | 81 |  |
| **Weight worries** |  |  |  |  |  |  |  |  |  |  |  |  |
| A lot | 14 | (6.2) | 208 | (18.8) | 314 | (20.6) | 75 | (29.0) | 412 | (38.3) | 694 | (43.7) |
| None / a bit | 212 | (93.8) | 899 | (81.2) | 1212 | (79.4) | 184 | (71.0) | 663 | (61.7) | 893 | (56.3) |
| Missing | 15 |  | 9 |  | 46 |  | 5 |  | 5 |  | 35 |  |
|  |  |  |  |  |  |  |  |  |  |  |  |  |
| **Total** | **241** |  | **1116** |  | **1572** |  | **264** |  | **1080** |  | **1622** |  |
|  |  |  |  |  |  |  |  |  |  |  |  |  |
