# Supplementary material for: Obesity among Scottish 15 year olds 1987–2006: prevalence and associations with socio-economic status, well-being and worries about weight
Source: BMC Public Health. 2008 Dec 9;8:404. doi: 10.1186/1471-2458-8-404 (PMC2615437; doi:10.1186/1471-2458-8-404)
Supplement: Additional file 2 — Table 2: Associations between obesity and socio-economic status (social class and area deprivation) – percentages (95% CIs) (and numbers) of males and females at each date. [file 1471-2458-8-404-S2.doc]

**Table 2: Associations between obesity and socio-economic status (social class and area deprivation) - percentages (95% CIs) (and numbers) of males and females at each date.**

|  |  |  |  |  |  |  |  |
| --- | --- | --- | --- | --- | --- | --- | --- |
|  | **Males** | | |  | **Females** | | |
|  |  |  |  |  |  |  |  |
|  | **1987** | **1999** | **2006** |  | **1987** | **1999** | **2006** |
|  |  |  |  |  |  |  |  |
| **Social class** |  |  |  |  |  |  |  |
| Non-manual | 9.1 (5.2-15.5)  (11/121) | 9.4 (7.1-12.3)  (47/501) | 15.2 (13.0-17.8)  (132/866) |  | 3.5 (1.4-8.6)  (4/115) | 10.0 (7.6-13.2)  (45/448) | 13.8 (11.6-16.4)  (108/781) |
| III-manual | 5.4 (2.1-13.1)  (4/74) | 11.2 (8.3-15.1)  (37/329) | 15.4 (11.9-19.8)  (50/324) |  | 6.2 (2.9-12.8)  (6/97) | 13.5 (10.1-17.9)  (40/296) | 15.1 (11.7-19.4)  (51/337) |
| IV-V | 2.3 (0.4-12.1)  (1/43) | 11.9 (8.2-17.2)  (24/201) | 16.7 (12.1-22.6)  (32/192) |  | 8.9 (3.5-20.7)  (4/45) | 12.2 (8.5-17.1)  (27/222) | 15.4 (11.2-20.9)  (33/214) |
| Missing | 0.0 (0.0-79.3)  (0/1) | 12.7 (6.6-23.1)  (8/63) | 20.1 (14.3-27.6)  (28/139) |  | 0.0 (0.0-49.0)  (0/4) | 10.6 (5.7-18.9)  (9/85) | 18.7 (13.5-25.3)  (31/166) |
|  |  |  |  |  |  |  |  |
| **Area deprivation category** |  |  |  |  |  |  |  |
| Low (categories 1-3) | 6.6 (3.2-13.0)  (7/106) | 8.9 (6.5-12.1)  (36/404) | 15.3 (12.7-18.3)  (99/648) |  | 0.9 (0.2-5.0)  (1/109) | 9.7 (7.1-13.1)  (36/371) | 12.0 (9.6-14.8)  (71/593) |
| Mid (categories 4-5) | 7.3 (3.6-14.3)  (7/96) | 11.7 (8.6-15.7)  (37/317) | 14.1 (11.5-17.2)  (80/566) |  | 4.8 (2.1-10.7)  (5/105) | 13.9 (10.7-18.1)  (47/337) | 17.2 (14.3-20.4)  (101/588) |
| High (categories 6-7) | 5.4 (1.5-17.7)  (2/37) | 12.6 (9.3-17.0)  (36/285) | 19.5 (15.4-24.4)  (58/297) |  | 17.0 (8.9-30.1)  (8/47) | 11.3 (8.2-15.4)  (33/292) | 15.9 (12.3-20.4)  (50/314) |
| Missing | - | 8.0 (3.9-15.5)  (7/88) | 50.0 (23.7-76.3)  (5/10) |  | - | 9.8 (4.3-21.0)  (5/51) | 33.0 (6.1-79.2)  (1/3) |
|  |  |  |  |  |  |  |  |
