# Supplementary material for: Obesity among Scottish 15 year olds 1987–2006: prevalence and associations with socio-economic status, well-being and worries about weight
Source: BMC Public Health. 2008 Dec 9;8:404. doi: 10.1186/1471-2458-8-404 (PMC2615437; doi:10.1186/1471-2458-8-404)
Supplement: Additional file 3 — Table 3: GHQ 'caseness', 'low' self-esteem and weight worries by obesity status – males and females at each date. [file 1471-2458-8-404-S3.doc]

**Table 3: GHQ ‘caseness’, ‘low’ self-esteem and weight worries by obesity status – males and females at each date.**

|  |  |  |  |  |  |  |  |
| --- | --- | --- | --- | --- | --- | --- | --- |
|  | **1987** | | **1999** | | **2006** | | **Significance of obesity by date interaction** |
|  |  |  |  |  |  |  |
|  | **Percent (95%CI)**  **(numbers)** | **ORs** | **Percent (95%CI)**  **(numbers)** | **ORs** | **Percent (95%CI)**  **(numbers)** | **ORs** |
| **Males** |  |  |  |  |  |  |  |
|  |  |  |  |  |  |  |  |
| **GHQ caseness** |  |  |  |  |  |  |  |
| Not obese | 12.8 (9.0-17.9)  (28/219) | 1.00 | 15.1 (12.9-17.5)  (143/950) | 1.00 | 21.7 (19.5-24.1)  (266/1226) | 1.00 | Obesity*date p = .994 |
| Obese | 12.5 (3.5-36.0)  (2/16) | 0.97 (0.21-4.52) | 15.7 (10.1-23.4)  (18/115) | 1.05 (0.61-1.78) | 22.0 (17.1-27.7)  (51/232) | 1.02 (0.72-1.43) |  |
|  |  |  |  |  |  |  |  |
| **‘Low’ self-esteem** |  |  |  |  |  |  |  |
| Not obese | 14.8 (10.6-20.3)  (30/203) | 1.00 | 12.3 (10.4-14.6)  (118/958) | 1.00 | 12.7 (10.9-14.6)  (154/1217) | 1.00 | Obesity*date p = .730 |
| Obese | 12.5 (3.5-36.0)  (2/16) | 0.82 (0.18-3.81) | 12.4 (7.5-19.7)  (14/113) | 1.01 (0.56-1.82) | 15.6 (11.5-20.8)  (36/231) | 1.27 (0.86-1.89) |  |
|  |  |  |  |  |  |  |  |
| **Weight worries** |  |  |  |  |  |  |  |
| Not obese | 5.8 (3.3-9.8)  (12/208) | 1.00 | 17.6 (15.3-20.1)  (171/971) | 1.00 | 18.8 (16.7-21.1)  (234/1245) | 1.00 | Obesity*date p = .869 |
| Obese | 12.5 (3.5-36.0)  (2/16) | 2.33 (0.47-11.47) | 29.6 (22.0-38.5)  (34/115) | 1.96 (1.27-3.03) | 28.8 (23.3-34.9)  (67/233) | 1.74 (1.27-2.39) |  |
| **Females** |  |  |  |  |  |  |  |
|  |  |  |  |  |  |  |  |
| **GHQ caseness** |  |  |  |  |  |  |  |
| Not obese | 18.0 (13.6-23.4)  (43/239) | 1.00 | 31.7 (28.8-34.8)  (289/911) | 1.00 | 43.6 (40.8-46.4)  (530/1216) | 1.00 | Obesity*date p = .462 |
| Obese | 28.6 (11.7-54.6)  (4/14) | 1.82 (0.55-6.09) | 33.9 (26.0-42.8)  (40/118) | 1.10 (0.73-1.66) | 41.3 (34.9-48.1)  (86/208) | 0.91 (0.68-1.23) |  |
|  |  |  |  |  |  |  |  |
| **‘Low’ self-esteem** |  |  |  |  |  |  |  |
| Not obese | 31.6 (26.1-37.8)  (75/237) | 1.00 | 31.6 (28.6-34.7)  (284/900) | 1.00 | 34.5 (31.9-37.2)  (419/1214) | 1.00 | Obesity*date p = .716 |
| Obese | 33.3 (13.8-60.9)  (4/12) | 1.08 (0.31-3.70) | 38.3 (30.1-47.3)  (46/120) | 1.35 (0.91-2.00) | 45.5 (38.9-52.2)  (96/211) | 1.58 (1.18-2.13) |  |
|  |  |  |  |  |  |  |  |
| **Weight worries** |  |  |  |  |  |  |  |
| Not obese | 28.0 (22.7-33.9)  (68/243) | 1.00 | 37.8 (34.7-40.9)  (350/927) | 1.00 | 40.1 (37.5-42.9)  (501/1248) | 1.00 | Obesity*date p = .068 |
| Obese | 46.2 (23.2-70.9)  (6/13) | 2.21 (0.72-6.80) | 43.3 (34.8-52.3)  (52/120) | 1.26 (0.86-1.85) | 59.8 (53.2-66.1)  (131/219) | 2.22 (1.66-2.97) |  |
|  |  |  |  |  |  |  |  |
